# Anterior cingulate cortex regulates pain catastrophizing-like behaviors in rats

**DOI:** 10.1186/s13041-023-01060-8

**Published:** 2023-10-13

**Authors:** Hyun Jung Jee, Elaine Zhu, Mengqi Sun, Weizhuo Liu, Qiaosheng Zhang, Jing Wang

**Affiliations:** 1 Department of Anesthesiology, Perioperative Care and Pain Medicine, New York University Grossman School of Medicine, New York, NY 10016 USA; 2 Interdisciplinary Pain Research Program, New York University Langone Health, New York, NY 10016 USA; 3 Department of Neuroscience and Physiology, New York University Grossman School of Medicine, New York, NY 10016 USA; 4 Neuroscience Institute, New York University Grossman School of Medicine, New York, NY 10016 USA

**Keywords:** ACC, Catastrophizing, Pain, CPA, Optogenetic, Single photon imaging

## Abstract

Negative pain expectation including pain catastrophizing is a well-known clinical phenomenon whereby patients amplify the aversive value of a painful or oftentimes even a similar, non-painful stimulus. Mechanisms of pain catastrophizing, however, remain elusive. Here, we modeled pain catastrophizing behavior in rats, and found that rats subjected to repeated noxious pin pricks on one paw demonstrated an aversive response to similar but non-noxious mechanical stimuli delivered to the contralateral paw. Optogenetic inhibition of pyramidal neuron activity in the anterior cingulate cortex (ACC) during the application of repetitive noxious pin pricks eliminated this catastrophizing behavior. Time-lapse calcium (Ca^2+^) imaging in the ACC further revealed an increase in spontaneous neural activity after the delivery of noxious stimuli. Together these results suggest that the experience of repeated noxious stimuli may drive hyperactivity in the ACC, causing increased avoidance of subthreshold stimuli, and that reducing this hyperactivity may play a role in treating pain catastrophizing.

**Supplementary Information:**

The online version contains supplementary material available at 10.1186/s13041-023-01060-8.

## Introduction

Pain is a complex sensory and affective experience, and its perception is strongly influenced by cognitive and emotional contexts [1]. While pain plays a physiologically vital role by alerting organisms of the potential for tissue damage [2], maladaptive responses to pain can negatively impact an individual’s quality of life. For example, anticipating the onset of pain can be an important adaptive behavior to prevent harm; pain catastrophizing, however, is a maladaptive coping behavior characterized by exaggerated negative affect when experiencing or anticipating pain [3], and is associated with enhanced postoperative pain and higher incidence of chronic pain [4–8]. Pain catastrophizing includes rumination, magnification of pain experience, and feelings of helplessness [9], and it is especially common in surgical patient populations [10]. Catastrophizing is also a risk factor for drug and alcohol dependence, predicting prolonged opioid use and misuse in postsurgical patients [11–14]. Various interventions, including patient education, physiotherapy, and cognitive behavioral therapy (CBT), have been used to reduce pain catastrophizing in patient populations, but only with limited effects [3, 15–19]. Thus, better understanding the neurobiological mechanisms of pain catastrophizing is crucial for developing effective therapeutic approaches to pain management.

The anterior cingulate cortex (ACC) is a key component of the cortical pain network involved in processing both acute and chronic pain [1, 2, 20, 21]. Nociceptive information is conveyed to the ACC through projections from the thalamus, amygdala, and other pain-related cortices such as the primary somatosensory cortex (S1) and insular cortex [2]. Increased activity in the ACC is associated with the processing of acute noxious inputs as well as processing of chronic pain [1, 20, 22–24]. For example, in human neuroimaging studies, patients suffering from chronic pain have demonstrated decreased gray matter in the ACC compared to healthy controls and recovered their gray matter volume after becoming pain-free [25].

ACC neurons are known to play a key role in the aversive, or affective, response to pain [26–32]. Neural activity in this region has been shown to decode the intensity and timing of pain [31, 33–37]. In rodents, inhibiting or lesioning the ACC has been found to result in decreased aversion to noxious stimuli in conditioned place aversion (CPA) assays [27, 31, 32, 38, 39]. These studies indicate that the ACC is necessary for encoding the aversive value of a noxious stimulus. Further, the projection from the ACC to the nucleus accumbens (NAc) mediates the social transfer of pain and analgesia, where mice demonstrate pain behaviors in the absence of external input when witnessing a peer receive painful stimuli and recover when the peer is given analgesia [40]. In addition to its role in aversive processing, the ACC is also involved in attention [41–44], decision making [45–47], and importantly, prediction of action and rewards [48–50], and these higher level functions further support its role in pain anticipation or catastrophizing.

Human fMRI studies have demonstrated a link between pain catastrophizing and ACC activity, where a decrease in pain catastrophizing is associated with changes in gray matter in the ACC [25, 51, 52]. It is, however, unclear whether directly manipulating the ACC can reduce or eliminate catastrophizing, as there is a lack of animal models that facilitate the causal investigation of this behavior. Here, we modeled pain catastrophizing behavior in rats and developed a novel behavioral assay, based on the CPA paradigm, to quantify this behavior. We found that animals that received repeated noxious pin pricks on one paw demonstrated an aversive response to non-noxious mechanical stimuli delivered to the opposite paw. Optogenetic inactivation of ACC pyramidal neuron activity during the delivery of repetitive noxious pin pricks eliminated this catastrophizing behavior. Time-lapse calcium (Ca^2+^) imaging in the ACC further revealed an increase in spontaneous neural activity after the delivery of noxious stimuli. These results suggest that the experience of repeated noxious stimuli may drive hyperactivity in the ACC, causing increased avoidance of subthreshold stimuli, and that reducing this hyperactivity may treat pain catastrophizing.

## Results

### Exposure to repeated noxious stimuli causes aversion to neutral sensory stimuli

**Fig. 1:**
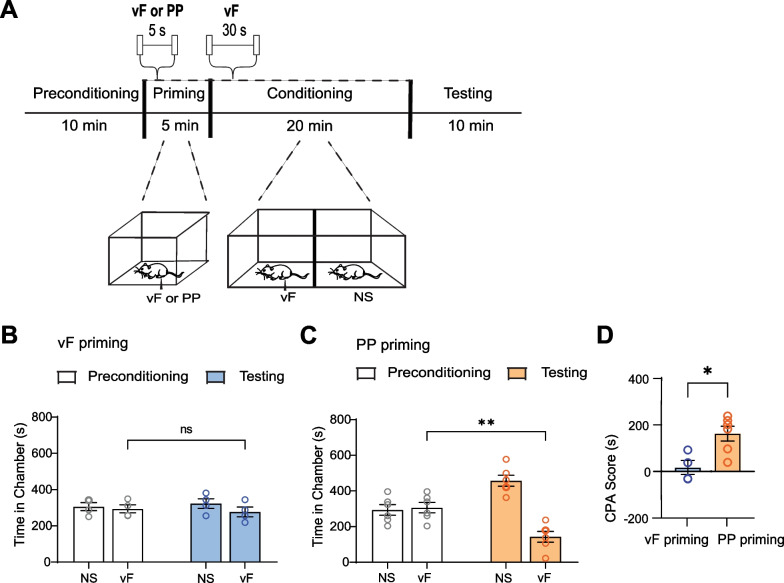
Repeated noxious mechanical stimuli induces aversion to non-noxious stimuli to mimic catastrophizing behavior. **A** Timeline of behavioral experiments, where either the noxious pin prick (PP) or the non-noxious von Frey (vF) stimulus was applied to the rat’s hind paw during priming, and the vF stimulus was delivered during conditioning. **B** Rats that were delivered the vF stimuli during priming exhibited no aversion to subsequent vF stimuli (p = 0.6105, paired t test; n = 4 animals) (Preconditioning NS: 306.2 ± 22.39, Preconditioning vF: 293.8 ± 22.39, Testing NS: 323.2 ± 26.50, Testing vF: 276.9 ± 26.50). **C** Rats that were delivered the PP stimuli during priming exhibited aversion to subsequent vF stimuli (**p < 0.01, paired t test; n = 6 animals) (Preconditioning NS: 293.8 ± 29.27, Preconditioning vF: 306.2 ± 29.26, Testing NS: 457.6 ± 30.42, Testing vF: 143.4 ± 30.16). **D** Rats primed with PP showed a greater aversion to the vF than did rats primed with vF (*p < 0.05, unpaired t test; n = 4 vF priming animals and n = 6 PP priming animals) (vF: 16.98 ± 29.95, PP: 162.8 ± 31.76). Data are represented as mean ± SEM

A key clinical feature of pain catastrophizing is enhanced aversive response to either a minimally noxious or non-noxious stimulus [9]. To model pain catastrophizing behavior in rodents, we developed a novel behavioral assay that involved nociceptive priming, followed by a classic conditioned place aversion (CPA) test. First, rats were placed in a two-chamber apparatus and were allowed to move freely between the chambers to establish non-preference for either treatment chamber during the preconditioning phase of the CPA assay. Next, during a priming phase, animals were removed from the CPA chambers and received repeated peripheral stimulation from a noxious 27G pin prick (PP) applied to one of their hind paws every 5 s for 5 min from under the same mesh table. This priming phase mimics a period of repetitive noxious stimulation that could potentially induce catastrophizing behaviors. As a control for priming with noxious stimulus, a non-noxious von Frey (vF) filament was applied during priming (Fig. 1A). Next, during the conditioning phase of the CPA assay, rats were transported back to the two-chamber apparatus, and one chamber was paired with repeated non-noxious mechanical stimulation with a vF filament delivered to the opposite paw, while the other chamber was not associated with any sensory stimuli (NS) (Fig. 1A). We hypothesized that priming with a noxious stimulus (PP) could produce catastrophizing-like behavior in response to a similar but non-noxious mechanical stimulus (vF) during the conditioning phase. During the testing phase, rats were again allowed to move freely between the two chambers without any stimulus.

Rats that received the non-noxious vF stimulation during the priming phase spent approximately equal amounts of time in the vF and NS chambers during the testing phase, showing no preference for either chamber (Fig. 1B). In contrast, animals that received the noxious PP during the priming phase avoided the chamber paired with vF stimulation (Fig. 1C), indicating that immediately prior exposure to painful stimuli (PP), but not neutral sensory stimuli (vF), caused rats to demonstrate an aversion to a similar mechano-sensory, albeit non-noxious, stimulus (vF). To quantify the difference between the two groups, we compared the CPA score of rats exposed to PP during the priming phase with that of rats exposed to vF during this phase. CPA scores were computed by subtracting the amount of time rats spent in the vF chamber during the testing phase from the time spent in the same chamber during the preconditioning phase. A higher CPA score indicates greater aversion to the vF chamber. We found that the group exposed to noxious PP stimuli during the priming phase had a statistically higher CPA score than the group exposed to vF (Fig. 1D), further demonstrating that PP-primed rats show greater aversion to neutral sensory stimuli than vF-primed rats. To further support these important pain-aversive findings in the context of catastrophizing, we measured peripheral sensitivity after priming. We found that rats that experienced priming with noxious stimulations demonstrated an increased tendency for paw withdrawals to non-noxious stimuli, compared with rats that did not receive noxious priming (Additional file 1: Fig. S1). Together, these results support a rodent behavioral model for pain catastrophizing, whereby prior exposure to pain results in negative expectation of pain and consequently abnormally enhanced aversive response to a neutral non-noxious peripheral stimulus.

### Inactivating the ACC eliminates catastrophizing-like behavior

**Fig. 2:**
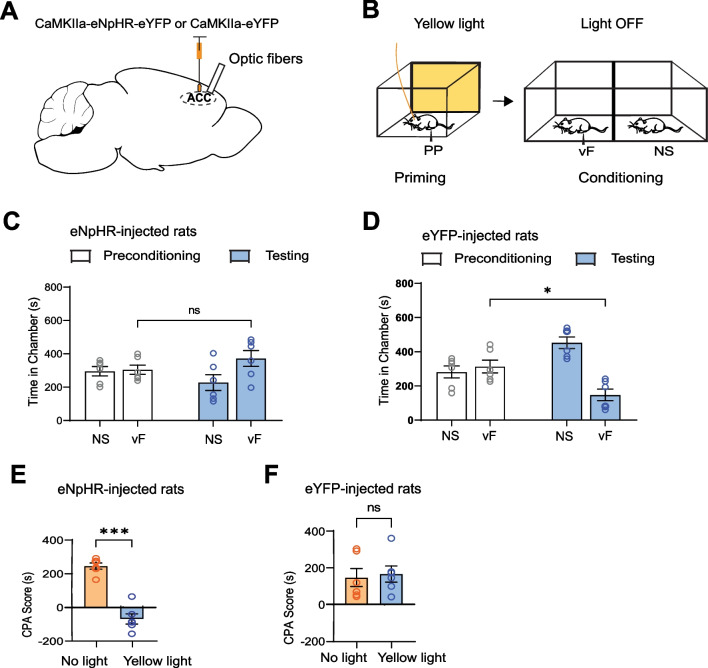
Optogenetic inhibition of the ACC during priming eliminates catastrophizing-like behavior. **A** Injection of AAV1.CaMKIIa.eNpHR.3.0.EYFP or AAV1.CaMKIIa.eYFP virus and optic fiber implantation into the ACC. **B** Behavioral assay of eNpHR and eYFP rats. Rats received yellow light treatment to the ACC during priming, while PP were delivered. No light was delivered during conditioning, where one chamber was paired with vF while the other chamber was paired with no stimulus (NS). **C** After yellow light treatment to the ACC during priming, eNpHR rats no longer showed an aversion to the vF chamber (p = 0.0766, paired t test; n = 6 animals) (Preconditioning NS: 295.9 ± 28.15, Preconditioning vF: 304.2 ± 28.15, Testing NS: 227.7 ± 47.24, Testing vF: 372.3 ± 47.24). **D** After yellow light treatment to the ACC during priming, eYFP rats still demonstrated an aversion to the vF chamber (*p < 0.05, paired t test; n = 6 animals) (Preconditioning NS: 281.6 ± 35.51, Preconditioning vF: 313.4 ± 37.57, Testing NS: 452.8 ± 33.58, Testing vF: 147.2 ± 33.58). **E** Yellow light treatment during priming reduced eNpHR rats’ aversion to the vF stimulus, relative to no light treatment (***p = 0.0001, paired t test; n = 6 animals) (No Light: 245.5 ± 18.73, Yellow Light: -68.17 ± 30.63). **F** Yellow light treatment during priming did not reduce eYFP rats’ aversion to the vF stimulus, compared to no light treatment (p = 0.7889, paired t test; n = 6 animals) (No Light: 146.6 ± 49.03, Yellow Light: 166.2 ± 44.27). Data are represented as mean ± SEM

Having shown that priming rats with noxious peripheral stimuli causes an aversion to non-noxious stimuli, thereby establishing a pain catastrophizing-like paradigm, we then studied the role of the ACC in this catastrophizing behavior. To examine whether optogenetic inactivation of neurons in the ACC could affect the catastrophizing phenotype, we injected the inhibitory opsin, eNpHR, versus the control viral vector, eYFP, into the ACC bilaterally, followed by implantation of optic fibers in the same area (Fig. 2A). We then delivered yellow light (589 nm) to the ACC to inhibit CaMKII-expressing pyramidal neurons during the priming phase of this behavioral paradigm (Fig. 2B).

First, we conducted the behavioral assay in eNpHR-injected rats and delivered yellow light to the ACC during priming, while the rats were being exposed to the PP. We found that inhibiting ACC pyramidal neuron activity during this priming period alone sufficiently removed the catastrophizing-like behavior, as eNpHR rats no longer demonstrated an aversive response to the vF chamber during the testing phase of the assay (Fig. 2C). To confirm that inactivation of CaMKII-expressing pyramidal neurons in the ACC, rather than light treatment itself, altered the catastrophizing behavior in rats, we repeated the same behavioral test in rats injected with eYFP. When these animals received yellow light treatment to the ACC during the priming phase, they continued to avoid the vF-paired chamber, demonstrating the expected catastrophizing response (Fig. 2D). CPA scores for eNpHR-injected and eYFP-injected rats further corroborated the effect of ACC inactivation on animals’ behavior. For eNpHR rats, the score for yellow light treatment during priming was statistically lower than that of the control condition, where no light treatment was given (Fig. 2E). The scores for eYFP rats revealed no difference in aversion between the yellow light treatment condition and the no light treatment condition (Fig. 2F), showing that eYFP-injected control rats did not respond behaviorally to yellow light.

**Fig. 3:**
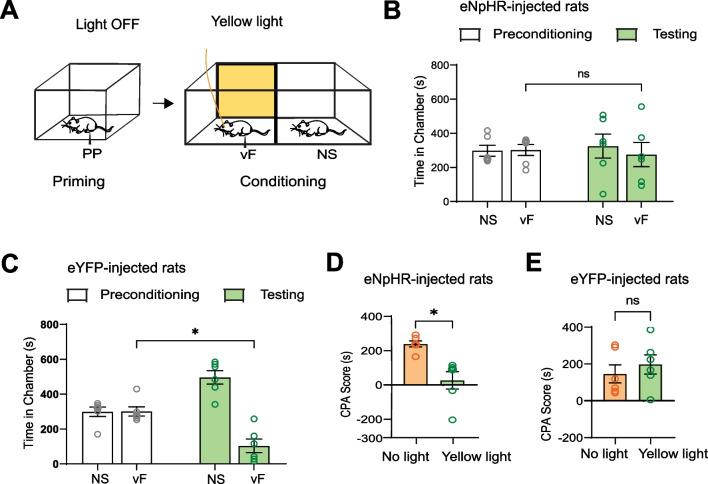
Optogenetic inhibition of the ACC during conditioning reduces catastrophizing behavior. **A** Behavioral assay of eNpHR and eYFP rats. Yellow light treatment was paired with the vF chamber during conditioning. **B** After yellow light treatment to the ACC during conditioning, eNpHR rats no longer showed an aversion to the vF chamber (p = 0.6251, paired t test; n = 6 animals) (Preconditioning NS: 298.0 ± 32.35, Preconditioning vF: 302.0 ± 32.35, Testing NS: 324.6 ± 70.36, Testing vF: 275.3 ± 70.36). **C** After yellow light treatment to the ACC during conditioning, eYFP rats continued to exhibit an aversion to the vF chamber (*p < 0.05, paired t test; n = 6 animals) (Preconditioning NS: 299.0 ± 26.53, Preconditioning vF: 301.0 ± 26.53, Testing NS: 496.5 ± 38.50, Testing vF: 103.5 ± 38.50). **D** Yellow light treatment during conditioning reduced eNpHR rats’ aversion to the vF stimulus, relative to no light treatment (*p < 0.05, paired t test; n = 6 animals) (No Light: 239.3 ± 17.89, Yellow Light: 26.58 ± 51.10). **E** Yellow light treatment during conditioning did not reduce eYFP rats’ aversion to the vF stimulus, compared to no light treatment (p = 0.4539, paired t test; n = 6 animals) (No Light: 146.6 ± 49.03, Yellow Light: 197.5 ± 52.27). Data are represented as mean ± SEM

ACC activities are known to be important for pain aversion, and thus as a control experiment, we treated the ACC with yellow light during the delivery of the vF stimulus in the conditioning phase (Fig. 3A). We found that for eNpHR-injected rats, ACC inhibition during the delivery of vF indeed removed the aversive value of vF, as expected from previous studies [31, 32] (Fig. 3B). In contrast, eYFP-injected rats continued to exhibit aversion to the vF chamber (Fig. 3C). CPA scores for eNpHR-injected and eYFP-injected rats further demonstrated the eNpHR rats’ behavioral changes in response to yellow light treatment. For eNpHR rats, the score for yellow light treatment during conditioning was lower than that of the control condition, where no light treatment was given (Fig. 3D), while the scores for eYFP rats showed no difference in aversion between the yellow light treatment condition and no light treatment condition (Fig. 3E). Importantly, for eNpHR rats, scores for ACC inhibition during priming or during conditioning were similar, suggesting that ACC activities are likely required for both the induction of catastrophizing and the processing of the actual aversive response to noxious stimuli.

### Hyperexcitability of ACC neurons after repeated exposure to noxious stimuli

**Fig. 4:**
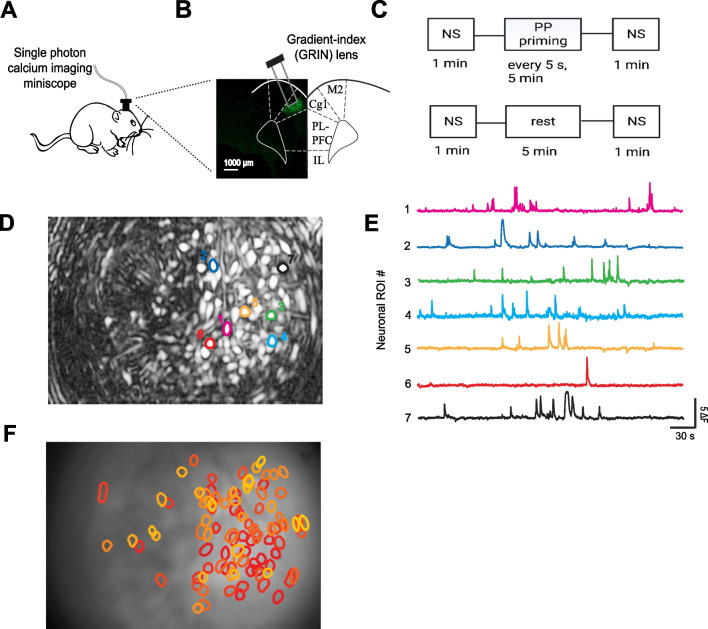
Schematic of in vivo endoscopic calcium imaging experiments. **A** Schematic of calcium imaging experiments. **B** Gradient-index (GRIN) lens placement and GCaMP6f expression in the ACC. **C** Timeline of calcium imaging experiments. **D** Field of view and sample identified contours of neuronal regions of interest (ROIs). (E) Calcium activity of neuronal ROIs identified in (**C**). **F** Map of ACC ROIs with contours overlaid on the imaging field of view

**Fig. 5:**
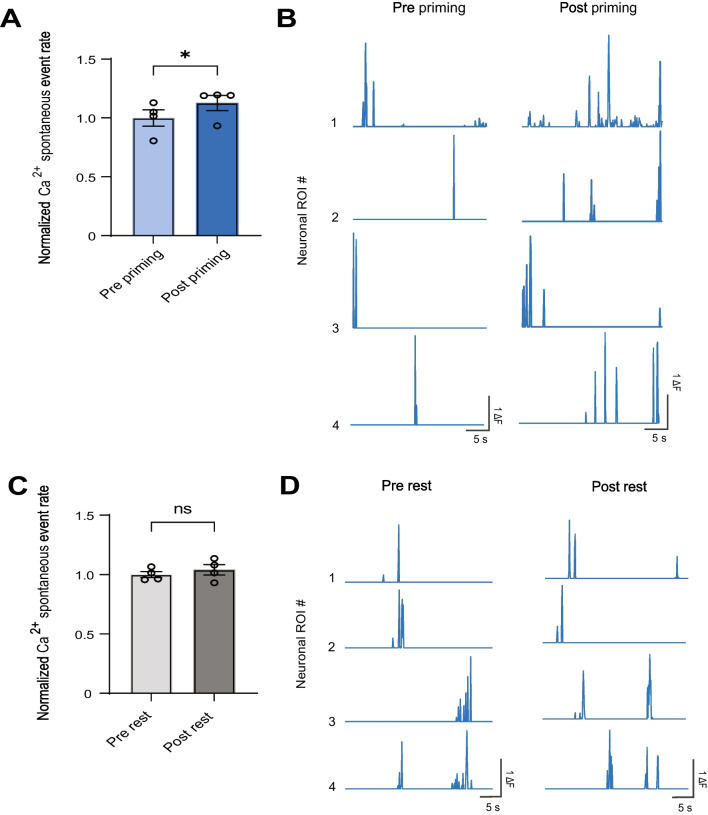
Priming with noxious stimuli increases calcium spontaneous activity in the ACC. **A** After priming rats with noxious PP, neuronal ROIs in the ACC showed increased calcium spontaneous activity (*p < 0.05, paired t test; n = 4 animals; 546 ROIs) (Pre-Priming: 1.000 ± 0.06921, Post-Priming: 1.128 ± 0.06472). **B** Representative calcium traces of neuronal ROIs before and after priming. **C** After undergoing 5 min of rest during priming, neuronal ROIs in the ACC showed no change in calcium spontaneous activity (*p* = 0.44, paired t test; n = 4 animals; 500 ROIs) (Pre-Priming: 1.000 ± 0.02547, Post-Priming: 1.040 ± 0.04429). **D** Representative calcium traces of neuronal ROIs before and after rest. Data are represented as mean ± SEM

As our CPA results show that inhibiting the ACC can reverse catastrophizing-like behavior, we then used time-lapse calcium (Ca^2+^) imaging in awake, freely-moving rats to characterize neuronal activity in the ACC before and after exposure to this noxious stimulus. We injected GCaMP6f and implanted a GRIN lens into the ACC, then mounted a single-photon miniscope (nVoke, Inscopix) above the lens to track Ca^2+^ activity within CaMKII-expressing pyramidal neurons (Fig. 4A, B). At the start of the imaging session, we measured spontaneous Ca^2+^ activity for 1 min. We then delivered the noxious PP stimulus for 5 min and recorded Ca^2+^ activity immediately after priming with the PP for 1 min. As a control for the PP session, we also measured Ca^2+^ activity without priming with PP (Fig. 4C). For each rat, we identified a population of neurons that were active during the imaging session (Fig. 4D–F), and then analyzed the Ca^2+^ spontaneous activity of these neurons for 30 s, both before and after exposure to the noxious PP. We found that after repeated exposure to the PP, the spontaneous Ca^2+^ activity for this neural population increased (Fig. 5A, B). In contrast, without priming, Ca^2+^ activity remained unchanged (Fig. 5C, D). These results suggest that priming with noxious stimuli likely drives hyperactivity in the ACC, which may in turn underlie catastrophizing behavior.

## Discussion

In this study, we developed a rat model to study pain catastrophizing. Acute repeated exposures to noxious stimulations produced an increased aversive response to subsequent non-noxious stimulus, even if that stimulus was delivered to another anatomic location. Furthermore, we showed that neurons in the ACC likely play a key role in this catastrophizing behavior, as inhibition of pyramidal neurons from this region prevented this pain catastrophizing-like behavior.

Although pain catastrophizing behavior is well characterized clinically [3–8, 10], there is a lack of animal models for it. Salient features of pain catastrophizing include prior pain experiences, expectation of future pain experience, and magnification of pain aversion [9]. In our model, we used repeated mechanical noxious stimulations to induce an immediately prior pain experience, and then measured expectation and magnification of pain aversive experience using the CPA test. Thus, our model incorporates several of the important features of pain catastrophizing. On the other hand, there are other features of pain catastrophizing which are not directly tested in our model, including rumination and feelings of helplessness [9]. Future studies are thus needed to assess rumination and helplessness in the context of pain in our model. Future studies should also include assessment of anxiety- and depression-like behaviors, which can often be found with pain catastrophizing behaviors clinically. These future studies could both further validate our model and explore additional dimensions of the relationship between pain, negative mood, and pain catastrophizing. Another limitation of our model is that we utilized noxious stimulations immediately prior to testing to induce catastrophizing. Clinically, however, catastrophizing can also be triggered by the memory of remote prior pain experience (from blood draw or a surgical procedure, etc.). Thus, future studies in animal models may also need to focus on analyzing remote pain memory in the context of a new potential pain experience.

In our study, we have shown that the ACC plays a critical role in catastrophizing-like behavior. Our in vivo imaging studies demonstrate an increase in the basal activity level of excitatory neurons in this region. Such findings are compatible with neuroimaging studies showing the activation of this region in the context of catastrophizing [25, 51, 52]. Interestingly, hyperactivity in the ACC is also observed in the context of chronic pain [31, 32, 53, 54]. Indeed, our experiments demonstrate that inhibiting the ACC can remove pain aversion, as expected from these previous studies [31, 32, 53, 54]. Thus, the ACC may play a role not only in the processing of aversive response to an acute noxious stimulus, but also in a variety of pathological situations such as chronic pain and catastrophizing. Importantly, our optogenetic experiments also indicate that inhibition of the ACC output neurons during the priming period—the period when catastrophizing is presumably induced—can also reduce catastrophizing behaviors. These results thus support a causal relationship between ACC activation and pain catastrophizing. At the same time, these results on the relationship between ACC activity and catastrophizing also reinforce the validity of our behavioral model.

In our study, the aversive response occurred after stimulation of the paw opposite to the one that received noxious priming. Thus, due to the non-somatotopic representation of this pain-aversive response and the relatively short period of priming, peripheral and spinal hypersensitivities are less likely causes than brain mechanisms of catastrophizing. However, future studies may be needed to further dissect peripheral and central contributions to catastrophizing behaviors. In our study, we were not able to distinctly resolve laminar or rostral-caudal locations of the ACC. Future studies utilizing two-photon imaging may best address this question to achieve better understanding of the role ACC plays in pain catastrophizing.

In conclusion, we have designed a rodent model for pain catastrophizing, and our results indicate that neurons in the ACC play a key role in this important pathological behavior. Future studies can further refine this behavioral model and analyze in greater detail the underlying cortical and subcortical mechanisms.

## Experimental model and subject details

### Animals

All procedures were performed in accordance with the New York University School of Medicine (NYUSOM) Institutional Animal Care and Use Committee (IACUC) guidelines to ensure minimal animal use and discomfort, as consistent with the National Institute of Health (NIH) *Guide for the Care and Use of Laboratory Animals*. Wild-type male Sprague–Dawley rats were purchased from Taconic Farms (Albany, NY) and housed at the vivarium facility in the NYU Langone Science Building under controlled humidity, temperature, and 12 h (6:30 AM to 6:30 PM) light–dark cycle. Food and water were provided ad libitum*.* All animals were about 7 weeks old upon arrival at the vivarium facility and were given 10–14 days to adjust to the new environment prior to any behavioral experiments or surgical procedures.

## Materials and methods

### Experimental protocol and data acquisition

All experimental studies were conducted in accordance with the New York University School of Medicine (NYUSOM) Institutional Animal Care and Use Committee (IACUC) regulations to ensure minimal animal use and discomfort, license reference number: IA16-01388. Male Sprague–Dawley rats were purchased from Taconic Farms and kept in a rearing room facility in the NYU Langone Science Building, controlled for humidity, temperature, and a 12-h (6:30 a.m. to 6:30 p.m.) light–dark cycle. Food and water were available ad libitum. Animals arrived at the facility weighing 250 to 300 g and had an average of 10 days to acclimate to the new environment before the experiment began.

### Virus construction and packaging

The recombinant adeno-associated virus (AAV) vectors were serotyped with AAV1 coat proteins and packaged at Addgene viral vector manufacturing facilities. Viral titers for pENN.AAV1.CamkII.GCaMP6f.WPRE.SV40, AAV1.CaMKIIa.eNpHR.3.0.EYFP, and AAV1.CaMKIIa.EYFP were approximately 5 × 10 [12] particles per milliliter.

### Intracranial viral injections and optic fiber implantation

Similar to previous studies [55, 56], rats were anesthetized with 1.5–2% isoflurane and were bilaterally injected with 0.65 μL viral vectors in the ACC. Injections occurred at a rate of 0.1 μL/20 s using a 32G 1 μL Hamilton syringe at anteroposterior (AP) + 3.2 mm, mediolateral (ML) ± 1.8 mm, and dorsoventral (DV) -2 mm, the syringe tips angled 30° toward the midline. After completing the injection, the microinjection needle was left in place for 10 min before it was raised 0.5 mm, allowing viral particles to diffuse and minimizing particle dispersion along the injection tract. The needle was held in place for an additional 5 min before being slowly raised from the brain. Rats were then implanted bilaterally with 200 μm optic fibers held in 2.5 mm ferrules (Thorlabs) at AP + 3.2 mm, ML ± 1.8 mm, and DV -1.5 mm, with the optic fiber tips angled 30° towards the midline. Dental acrylic was used to keep the optic fibers and ferrules in place. After intracranial injections and fiber implantation, rats were placed on a heating pad until their recovery from anesthesia and were monitored twice a day for any signs of pain or infection for approximately 3 days. Afterwards, animals were monitored once a day and were allowed to recover from their surgical procedure for about 4 weeks before starting behavioral experiments. For rats subjected to the gradient-index (GRIN) lens implantation, 0.65 μL of the GCaMP6f viral vector was injected unilaterally into the ACC at AP + 2.9 mm, ML ± 1.6 mm, and DV -2 mm, with the syringe tip angled 22° towards the midline. After surgery, the rats were placed on a warm pad until their recovery from anesthesia and were monitored twice per day for 3 days to prevent any infection or pain. We waited approximately 4 weeks to express the virus properly before the GRIN lens implantation procedure.

### Gradient-index lens implantation and mounting

4–6 weeks after the intracranial injection of the GCaMP6f virus, rats were anesthetized with 1.5%–2% isoflurane and stereotaxically implanted with the gradient-index (GRIN) lens (1.0 mm diameter, ~ 9.0 mm length, Inscopix) at AP + 2.9 mm, ML ± 1.6 mm, and DV -1.8 mm, with the tip of the lens angled 22° towards the midline. The space between the lens and the site of the open craniotomy was filled by silicone elastomer (Kwik-Sil, World Precision Instruments). Dental acrylic was used to hold the lens in place. A piece of aluminum foil was used to cover the lens top and extra silicone elastomer was applied on top to the lens to protect the lens and prevent any debris. Animals were monitored for signs of pain or infection and were allowed to recover from their surgical procedure for about two weeks.

Approximately two weeks after the implantation of the GRIN lens, rats were anesthetized with 0.5–1% isoflurane and were inspected for GCaMP6f fluorescence and Ca^2+^ transient activity. The miniature microscope (nVoke, Inscopix) was attached to a baseplate and was stereotaxically adjusted relative to the location of the lens implant to determine an optimal field of view (FOV) for neural activity imaging. Both auditory (clapping) and sensory (tail pinching) stimuli were used to elicit neural activity, and a baseplate was mounted above the lens for rats that exhibited a Ca^2+^ response. After confirming the placement of the baseplate, the anesthesia was raised to 1.5–2% isoflurane, and the baseplate was held in place with adhesive cement (Metabond Quick! Adhesive Cement System, C&B). To protect the lens when not in use, a baseplate cover (Inscopix) was magnetically attached to the baseplate.

### GRIN lens imaging procedure

As described in previous studies [31, 56, 57], the rat was placed in a recording chamber over a mesh table at the beginning of the imaging procedure. The miniature microscope was mounted on the baseplate, with the FOV aligned as closely as possible to the previous recording’s FOV. The rat was allowed to habituate to the chamber for about 10 min before the start of each recording. During the imaging session, spontaneous neural activity was first recorded for 1 min while the rat moved freely within the chamber without any stimulus from the experimenter. Noxious peripheral stimulation was delivered to the plantar surface of the hind paw ipsilateral to the brain recording site by using a 27G needle pin prick (PP). Noxious stimulation was terminated upon withdrawal of the paw. For each recording session, the noxious stimulus was delivered every 5 s for 5 min. After completing the delivery of the noxious stimulus, rats’ spontaneous neural activity was again recorded for 1 min. Experiments were recorded by a video camera (HC-V550, Panasonic). No physical damage to the paws was observed.

### GRIN lens data acquisition and preprocessing

All miniature fluorescent microscope videos were recorded at a frame rate of 20 Hz, with a laser power of 0.6–0.8mW/mm^2^. Using the Inscopix Data Processing Software (Inscopix), raw videos were downsampled spatially by a binning factor of 4 (16 × spatial downsample) and temporally by a binning factor of 2 (down to 10 frames per second). After downsampling, the videos were motion-corrected relative to a single reference frame to match the XY positions of each frame throughout the video using the Inscopix Data Processing Software. The motion-corrected 10 Hz video of raw Ca^2+^ activity was saved as a.TIFF file and was used to for cell identification. Using modified constrained non-negative matrix factorization scripts (CNMF_E) in MATLAB, Ca^2+^ signals were extracted to estimate temporally constrained instances of calcium activity for each neuronal region of interest (ROI).

### Analysis of spontaneous calcium response

Spontaneous Ca^2+^ activity before and after priming with noxious PP stimulation was calculated as the mean event rate, similarly to previous studies [56, 58]. We took 30 s of the baseline recordings for spontaneous neural activity, both immediately before and after PP priming, and calculated a sliding median with a window of 4 s to remove fluctuations within the recordings. This median was then subtracted from the raw activity trace to obtain the processed trace, which was used to identify peaks—transient events that were greater than 2.5 SD above the baseline noise. Peaks with an inter-event time of < 2 s (or 20 frames) were removed. Using the number of peaks, we calculated for each neuron the mean Ca^2+^ transient event rate during the baseline periods before and after priming. To compute the mean Ca^2+^ transient event rate for each rat, we took the mean spontaneous rate of all neurons that were active during the imaging session. To compare spontaneous event rate before and after priming, we calculated the mean spontaneous event rate for all rats before priming and used this value to normalize the individual event rates for each rat both before and after priming.

### Behavioral assay

Our catastrophizing behavioral assay was developed based on the conditioned place aversion (CPA) test. At the beginning of the assay, the rat was placed in a two-chamber apparatus consisting of equally sized compartments, which were connected by a large opening that allowed free movement between the chambers. A different scented balm was applied to the walls of each chamber to provide the rat with contextual cues. The behavioral paradigm consisted of preconditioning (baseline), priming, conditioning, and testing phases. During the preconditioning phase (10 min), the rat was allowed to roam freely between the two chambers without any stimulus from the experimenter. Animals that spent more than 480 s or less than 120 s of the total time in either chamber during this phase was eliminated from further analysis. Immediately after the preconditioning phase, the rat was moved to a smaller single-chamber apparatus and underwent priming. The priming chamber was paired with noxious PP stimulation with a 27G needle, delivered to the plantar surface of the rat’s hind paw every 5 s for the duration of 5 min. During the conditioning phase (20 min), the rat was transported back to the two-chamber apparatus. One chamber was paired with a vF stimulus, delivered to the plantar surface of the hind paw opposite to the one used during the priming phase, and the other chamber was paired with no peripheral stimulus (NS). The order of the vF stimulus and the NS were counterbalanced, such that half of the rats received the vF stimulation first, while the other half received NS first during conditioning. Chamber pairings were also counterbalanced. During the testing phase (10 min), no stimulation was given by the experimenter, and the rat was allowed to travel freely between the two chambers. AnyMaze software and a video camera were used to track the movements of the rat in each chamber. Decreased time spent in a chamber during the testing phase compared to the preconditioning phase indicated avoidance (aversion) of that chamber, while increased time in a chamber indicated a preference for that chamber. The CPA score, which quantifies an animal’s aversion to the stimulus, was computed by subtracting the time the rat spent in the chamber associated with the vF stimulation during the testing phase from the time it spent in the same chamber during the preconditioning phase. A higher CPA score indicated greater aversion to the vF stimulus.

### In vivo optogenetic stimulation

Light at 589 nm wavelength was delivered bilaterally through optic fibers implanted in the rat brain, using a yellow diode pumped solid state (DPSS, Shanghai Dream Laser) laser. The laser was first connected to a rotary joint (Doric), mounted over the testing chamber, via a fiber optic patch cable. Two fiber optic patch cables attached to the rotary joint were connected to optic fiber cannulas on the rat's head through mating sleeves (ADAF1, Thorlabs). Laser output was controlled through TTL Pulse Generators (OPTG 4, Doric) and continuous laser light was delivered at the intensity of 5–6 mW at the fiber tip. The power output of the optic fiber tip was calibrated prior to each experiment.

### Supplementary Information

**Additional file 1.** Peripheral sensitivity to non-noxious stimuli is observed after priming with noxious stimuli.

## Data Availability

Data associated with this study are present in the paper or available upon reasonable request.
