# Supplementary material for: Anterior cingulate cortex regulates pain catastrophizing-like behaviors in rats
Source: Mol Brain. 2023 Oct 13;16:71. doi: 10.1186/s13041-023-01060-8 (PMC10576271; doi:10.1186/s13041-023-01060-8)
Supplement: Supplementary file 1 — Additional file 1. Peripheral sensitivity to non-noxious stimuli is observed after priming with noxious stimuli. [file 13041_2023_1060_MOESM1_ESM.pdf]

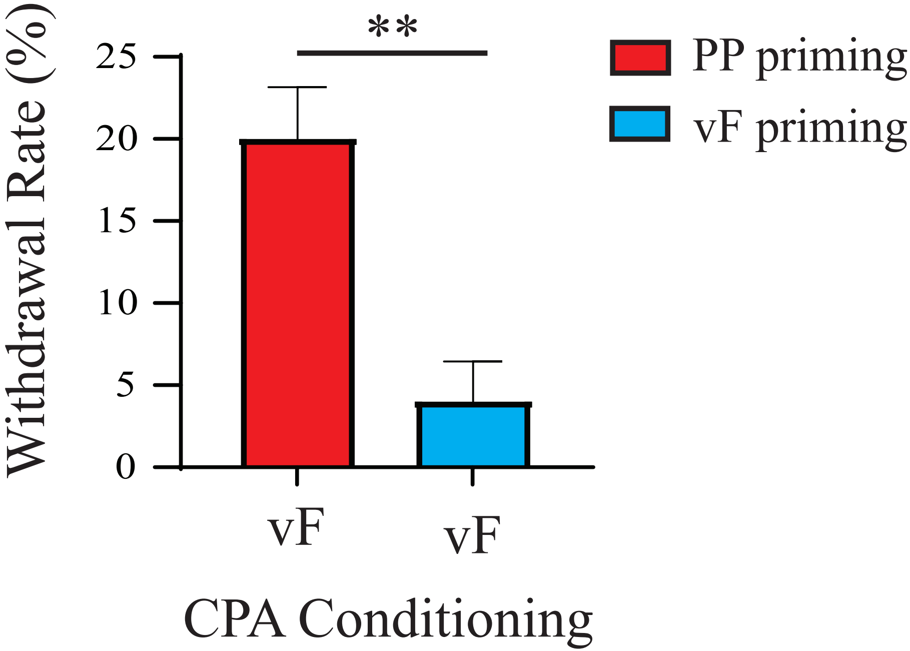

Additional file 1: Fig. S1. Peripheral sensitivity to non-noxious stimuli is observed after priming with noxious stimuli. After priming with a noxious stimulus (pin prick, or PP), rats withdrew their opposite paws to the non-noxious stimulus (vF) at a rate that is substantially higher than control rats (**$p<0.01$, paired t test; n = 5 animals).
